# Co-creativity, well-being and agency: A case study analysis of a co-creative arts group for people with dementia

**DOI:** 10.1016/j.jaging.2019.03.002

**Published:** 2019-06

**Authors:** Hannah Zeilig, Victoria Tischler, Millie van der Byl Williams, Julian West, Sarah Strohmaier

**Affiliations:** a London College of Fashion, University of the Arts, 20 John Prince's Street, London W1G 0BJ, United Kingdom; b College of Nursing, Midwifery and Healthcare, University of West London, St Mary's Rd, London W5 5RF, United Kingdom; c Royal Academy of Music, Marylebone Road, London N1 5HT, United Kingdom; d Salomons Centre for Applied Psychology, Canterbury Christ Church University, 1 Meadow Road, Tunbridge Wells, Kent TN1 2YG, United Kingdom

**Keywords:** Dementia, Arts, Co-creativity, Well-being, Agency

## Abstract

At the heart of this paper is an exploration of artistic co-creativity involving people with dementia and their partners. Co-creativity promotes a relational approach to creativity which nurtures inclusion and participation. This paper investigates how co-creativity can affect well-being from the perspectives of people with dementia and their carers; and explores how well-being and agency might be usefully reconsidered. The article draws on findings from a small-scale study ‘With All’ that focused on music and dance as non-verbal and therefore inclusive artforms. A range of disciplinary perspectives, from psychology, philosophy and social sciences, inform the study. The research used an intrinsic case-study methodology and within this a mixed-methods approach was adopted. This included dialogic interviews, video data analysis and the Canterbury Well-being Scale (CWS). Thematic analysis of the interviews and video data revealed three key themes: autonomy, connections, and art as an enabler. These themes captured the experiences of the participants and facilitated a more nuanced understanding of wellbeing and agency in the context of living with dementia. The analysis of the CWS indicated some improvements in well-being. Following this analysis using multiple data sources, the paper argues that well-being and agency are best understood as relational, and ongoing, rather than completed states. Further both wellbeing and agency contain their opposites (ill-being and passivity). This innovative exploration highlighted the importance of co-creative collaboration as a method that was considered valuable by participants, and that therefore should be further considered in future research with people living with dementia.

## Highlights

• Co-creativity is an inclusive method.
• Co-creativity is valued by people with dementia
• Agency and well-being are linked.
• Agency and well-being are relational.

## Introduction

This paper draws on data from ‘With All’, a co-creative arts project that took place over four weeks at the Wellcome Hub in London. In this project, people with dementia and their partners collaborated with musicians and dancers. Our aim is to elucidate the distinctive qualities of a co-creative approach and its possibilities for enhancing well-being and supporting agency. There is a scarcity of arts and health research that engages with the opinions and experiences of people with dementia and equally a tendency to assume that people with dementia are submissive recipients of the arts. As a means of contextualising the With All Study, the role of the arts for people with dementia is discussed before introducing the novel concept of co-creativity. Perspectives on wellbeing and agency are then outlined as these are relevant to the arts and for people with dementia. Finally, we present the methods and findings and discuss how the views of people with dementia and their partners can help us consider concepts (such as creativity, wellbeing and agency) afresh.

We adopt a transdisciplinary approach in which multiple perspectives, including those from the arts, philosophy, psychology and the social sciences are used to address a common question (Toomey et al., 2015:1). Transdisciplinary approaches are differentiated by their inclusion of knowledge and reflections from individuals outside academia (Mobjörk, 2010: 869). This is pertinent here, as the voices of people with dementia and their partners are central feature of this study.

### Dementia, the arts and co-creativity

The positive effect of the arts for people with dementia is largely uncontested. A growing body of international evidence documents the potential of the arts to impact positively on the health and well-being of people living with dementia (All-Party Parliamentary Group on Arts, Health and Wellbeing (APPGAHW), 2017, Camic, Crutch, and Zeilig (2018), Young, Camic, & Tischler, 2016, National Institute for Health and Care Excellence (NICE), 2015). There has also been a burgeoning awareness that arts-based research methods offer alternative insights into the subjective experiences of people living with dementia (Crutch, Isaacs, & Rossor, 2001; Sauer, Fopma-Loy, Kinney, & Lokon, 2014). However, the views of people with dementia about arts practices that they have been involved with, are rarely an integral part of research and consequently cannot inform future practice or the development of theory (Beard, 2004; Windle et al., 2017).

The purpose of many participative arts projects is predominantly to promote health, well-being, cognitive function and communication and therefore they tend to focus on instrumental benefits for people living with dementia. Within a cultural context that is dominated by a biomedical ethos (Krishna, 2014) a ‘dose of the arts’ is commonly given in measured amounts to people, as if the arts are carefully calibrated medications. Indeed, a recent report identified the ‘active ingredients’ of arts and health activities (Aesop, BOP, 2018). Within this paradigm, people with dementia are firmly located as passive ‘objects of study’ in receipt of specially designed arts interventions that can enhance health and well-being. This is quite different from deploying the arts as a means of engaging with and nurturing the innate creativity of people with dementia. Our work is inspired by the theory of musicking that explicitly includes dancing (Small, 1998). This theory emphasises the relational nature of music and dance as processes and states:

*"The act of musicking establishes in the place where it is happening a set of relationships, and it is in those relationships that the meaning of the act lies.”*

(Small, 1998:13)

Therefore, our work emphasises co-creativity as a relational practice that exists within a group.

The tendency to overlook the purely creative possibilities of the arts for those with dementia is connected with entrenched ideas about what creativity is, where it is located and how it is manifested (Camic, Crutch, Murphy, et al., 2018, Zeilig, West, & van der Byl Williams, 2018). For instance, from a neurological perspective, creativity has previously been linked to processes and outcomes correlated with individual motivation and stresses cognitive features (Palmiero, Di Giacomo, & Passafiume, 2012). This emphasis may be problematic for people with dementia who experience decline in cognitive processes and often increased interdependency. Even within less rigidly neurological parameters, there is a persistent assumption that people with dementia cannot be creative (Bellass et al., 2018). When creativity is acknowledged, it is conventionally as a way of understanding the underlying brain pathology of an individual (Crutch et al., 2001).

Co-creativity represents a novel way of understanding and collaborating with people with dementia that emphasises the shared and relational qualities of creativity. Despite the absence of a single agreed definition of ‘co-creativity’ its key features have been identified (Schmöelz, 2017; Zeilig et al., 2018). These include: a focus on shared process, shared ownership, inclusivity, reciprocity and relationality (Zeilig et al., 2018). Co-creativity necessitates and creates openness, equality and imaginative space. Above all, it contrasts with restrictive notions of the lone creative ‘genius’ that have tended to dominate views of creativity (Camic, Crutch, Murphy, et al., 2018). Co-creativity has been explored in relation to storytelling and playful activities amongst children in the classroom and is similarly described in this context as:

‘a process that integrates individual, collaborative as well as communal aspects of creativity’

(Schmöelz, 2017)

The With All project was subsequently designed in order to explore the possibilities of co-creativity with people with dementia and their partners.

### Towards conceptualising well-being and agency

Well-being has been understood by ancient philosophers as comprising both hedonic and eudemonic elements (Aristotle) and it is only in the mid-twentieth century that subjective experiences of well-being have been studied (Shin & Johnson, 1978). More recently, perspectives from positive psychology have emerged as a source of evidence for the value of well-being (Seligman, 2012) and improving well-being is an imperative that guides much public health and social policy (Office for National Statistics, 2018). Agency is also an important concept in relation to people with dementia and has been considered as ‘crucial’ to the understanding of wellbeing and person centred care (Chung et al., 2017, p.1).

#### Agency

Agency at its most broad is the idea of meaningful intentional action (Schlosser, 2015). It is connected with approaches to dementia that are based on human rights and citizenship (Bartlett & O'Connor, 2010; Kontos, Miller, & Kontos, 2017; Shakespeare, Zeilig, & Mittler, 2017), an area of increasing interest in dementia care (see e.g. Hughes & Williamson, 2019). The capacity to act and to effect change in the external world is a fundamental part of personhood. The standard association of agency with the capacity to act intentionally and the entrenched belief that the progress of dementia leaves people largely incapable of intentional, meaningful action has resulted in the assumption that dementia necessarily involves a loss of agency (Aquilina & Hughes, 2005; Jaworska, 1999; Jennings, 2010; Kontos et al., 2017). In a recent study a group of people with dementia discussed how on revealing their diagnosis, they were subsequently ‘denied an opportunity to contribute to society in a meaningful way’ (O'Connor, Mann, & Wiersma, 2018:50). The systemic assumption that people with dementia can no longer ‘do’ or ‘act’ necessarily results in their citizenship being challenged. This is reinforced by representations in the media and other cultural outputs e.g. film that link dementia to a lack of agency (Burke, 2017; Zeilig, 2014).

In addition, agency tends to be understood in individualistic terms (Boyle, 2014: 1131) and there is consequent tendency to regard increasing dependence on others as resulting in a loss of agency (Jennings, 2010:433). However, human beings are essentially socially embedded (Taylor, 1992) and interdependent, therefore agency (like well-being) must also be understood for people with dementia and all of us, as relational and influenced by sociocultural contexts. The notion of agency is pertinent in this study as co-creative approaches promote and encourage the involvement of people with dementia and challenge the passive roles that they are often ascribed (Kolanowski & Buettner, 2008:3).

#### Well-being

The concept of well-being in relation to those living with dementia has been considered by Kaufmann and Engel (2016) who extended Kitwood's five domain model (Kitwood & Bredin, 1992) by adding agency as a sixth domain to highlight the importance of subjective well-being in the dementias. A recent definition of well-being, informed by people with dementia, understands it as “a fluctuating subjective state [which] involves a sense of agency, engagement, happiness, feeling well, confidence and optimism” (Strohmaier & Camic, 2017). This definition is closely linked with the Canterbury Well-being Scale (CWS) used in this study. However, despite the development of scales and questionnaires, there is no consensus on how to measure well-being or even how to conceptualise it.

Although there has been an increasing interest in exploring well-being for people with dementia in arts projects (Gross, Danilova, & Vandehey, 2015, Osman, Tischler, & Schneider, 2016, Windle et al., 2017), there is a dearth of research that has focused on the capacity for agency in these contexts. This is understandable given a contemporary context that ‘gives’ the arts to people with dementia and assumes that dementia necessarily involves a loss of self and identity (Hughes et al., 2005). Most discussions or measures of either well-being or agency in relation to dementia start from a perspective that assumes absence or loss.

We are interested in how, through co-creativity, and from the perspective of people with dementia and their partners, we can begin to reconceptualise well-being and agency as they are closely interrelated, embodied and relational. We argue that both well-being and agency can be understood as ongoing, social practices rather than completed states. This work contributes to the relatively small body of research on agency and dementia and the more extensive literature on dementia, the arts and well-being.

## Methods

The project utilised a case study approach, characterised by iterative dialogue, reflective discussions following each project session. This facilitated a collaborative, multi-layered and in-depth investigation. Two of the authors (anon) did not take part in the co-creative sessions and therefore had a degree of detachment that helped to minimise bias in the analysis. The methodology is primarily qualitative but some quantitative analysis is included.

Case studies are recognised as being of particular value when understanding the experience of dementia (Hellström, Nolan, & Lundh, 2005). This is because the case study method captures the complexity of real-life events while maintaining a holistic understanding (Yin, 1994:3). Moreover, a case-study approach was particularly apt due to the exploratory and explanatory nature of our research (Yin, 1994:6). We used an intrinsic case study approach, focusing on one unique phenomenon, due to the originality of the With All project (Crowe et al., 2011). However, the intrinsic case study approach has also enabled us to draw conclusions that will be of broader significance to other arts activities and to wider theoretical and conceptual discussions around those living with dementia (Hellström et al., 2005:12).

The case study comprised 4 × 1 h co-creative group arts sessions that took place weekly at the Hub at the Wellcome Collection over a 4-week period. The refreshments were offered in the entrance area of the Hub and the room in which the activities took place was arranged with a large circle of chairs round the outside of the room with a table of instruments easily accessible at one end. The group comprised three musicians (an oboist, a cellist and a percussionist), two dancers, two researchers, five people living with dementia and three partners. The attendance of people with dementia and their partners varied as a result of ill-health, but was never less than three people with dementia and two partners. All were able to give informed consent. Ethical approval was granted by UCL Ethics Committee (Approval number 8545/002). Due to ethical constraints, it was not possible to involve people with more advanced dementia.

The artists involved were experienced in improvisatory music and all had worked with people with dementia. We held a meeting with the artists in September 2017, before the start of the project, to discuss what we understood by co-creativity, and the ways in which the artists had experience working in these ways. Musical instruments were lent to the project by the Royal Academy of Music and included hand chimes, tambourines and drums, alongside more unusual instruments from the teams' personal collections including Baoding balls and Kalimbas. The instruments were selected because they did not require highly developed technical skills in order to achieve a satisfactory musical effect. Music and dance were used as they can be largely non-verbal, thus enabling communication without language and facilitating the participation of those with dementia. In addition, music and dance more easily facilitate spontaneous collaboration, with a focus on process rather than product (Levinson, 2015:164).

The research team worked in collaboration with several organisations to invite people to participate, including Rare Dementia Support Groups (UCL), Resonate Arts, and a local branch of the Dementia Engagement and Empowerment Project (DEEP). People with dementia were consulted at the inception of the project in December 2017 about its design and methods. In line with their comments, less formal interview methods were used and refreshments were included at the beginning and end of each session – so that there were ample opportunities to socialise.

Collecting multiple sources of data (both qualitative and quantitative) increases the validity of a case study (Crowe et al., 2011, p6). Thus, we used dialogic interviews, video data and the Canterbury Well-Being scale (CWS) in an effort to capture the complexity of these sessions and the experience of people with dementia and partners. We also recorded field notes as supportive documents rather than as objects of study.

### Code and theme development

The development of a coding approach for both the interview transcripts and the video data benefitted from iterative cycles of work across a variety of data sources, distributed expertise and the involvement of a non-biased researcher (anon). Braun and Clarke's (2006) structured approach influenced thematic analysis of video and interview data. This involved a six phase process including familiarisation with data, coding, thematic search, naming and defining themes, and writing up. Analysis combined deductive and inductive processes. For example, the authors were interested in participant agency therefore this warranted a deductive approach. The analysis also used induction to identify, for example, potential dis-benefits of co-creative processes. Through iterative coding and discussion, the team collapsed the codes into code groups and then further into three main themes which are discussed below.

## Findings

The findings are discussed in two sections. The first (3.1) describes the findings from the thematic analysis, verbatim quotes are used to illustrate the thematic findings, with pseudonyms assigned to protect anonymity. The second (3.2) presents results from the Canterbury Well-Being Scale.

### Thematic analysis

**Fig. 1:**
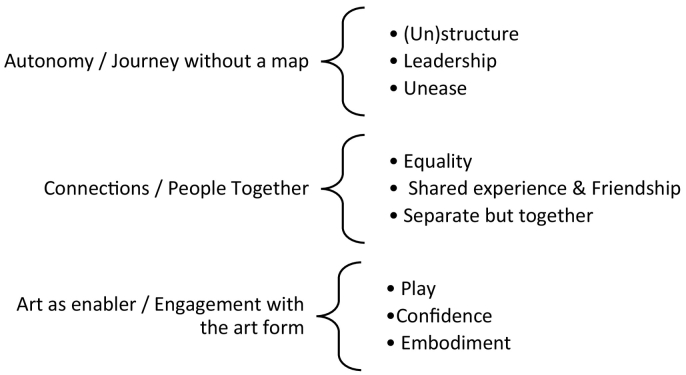
Super and subordinate themes.

Fig. 1 below outlines the relationship between the super and subordinate themes (that are discussed in detail below). These themes were identified after rigorous engagement with the data, following a systematic thematic analysis of the video and interview data involving all authors. (See Table 1.)

**Table 1:** Case study methods and characteristics.

Data	Data collection	Data analysis
Interviews	Collected by…anon for review Audio-recorded, dialogic interviews were used (sessions 8, 9 and 10). A total of 8 interviews took place, 5 with people with dementia and 3 with their partners. Dialogic interviews are interactive and are not simply descriptive but co-construct new narratives (Russel & Kelly, 2002). Thus, research results are shaped by both researcher and respondent. The dialogic interviews lasted for approximately 30 min. These were carried out by researchers experienced in working with people with dementia	Coded by…anon for review All interviews were digitally recorded and transcribed verbatim to ensure the credibility and confirmability of data collection. Interviewees were given the opportunity to review and revise these transcripts. They were then coded iteratively by 4 of the authors (anon) using Atlas.ti software – incidents, views and experiences were compared across the interview data. In order to explore the commonalities of experience of participants, a thematic analysis (Braun & Clarke, 2006) was carried out across all the interviews.
Video data	Collected by… anon for review Each session was video recorded by two cameras: positioned at opposite ends of the room to capture interactions from several angles. We used the video to gather further data, especially non-verbal elements of the sessions as well as interactions and key moments.	Coded by … anon for review Our detailed, observational field notes (see above) were used as a way of indexing the data. We adopted an inductive, discovery orientated approach to coding the video data. Following Derry (2007: 22) 4 authors (anon) viewed the unedited corpus of the video in its entirety, as a means of identifying major events and investigating our broad questions about agency and well-being. All 4 researchers coded all 4 sessions using Atlas.ti simultaneously as suggested by (Saldaña (2016): 63). This ensured the “confirmability and trustworthiness of findings”(ibid), but also enabled us to discuss our reflections after each session. The directness of this approach enabled us to stay close to the data.
Canterbury Wellbeing Scale (Johnson, Culverwell, Hulbert, Robertson, & Camic, 2017)	Collected by… anon for review The Canterbury Well-being Scale (CWS) was used to gauge participant well-being before and after taking part in With All. The scales were completed at the beginning and end of each session by people with dementia. The CWS are visual analogue scales measuring how interested, confident, optimistic, happy and well participants feel.	Statistical Analysis (see below)
Field Notes	Collected by…anon for review Field notes were taken during the sessions by anon and anon using a template. anon also made notes immediately after the sessions.	Coded by …anon for review These field notes were put into Atlas.ti and coded alongside the interviews and video data. They were used only as supportive data for the video analysis rather than as objects of study in themselves.

**Fig. 2:**
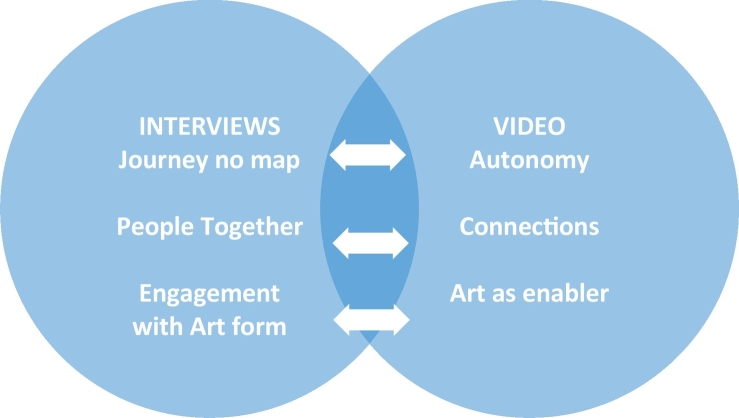
Relationship between themes: interview and video data.

As outlined in Fig. 2 below, the themes overlap and are therefore discussed jointly.

#### Journey with no map/autonomy

The co-creative sessions were based on improvisation and a consequent lack of preconceptions or expectations. Therefore, there was no predetermined sense of direction. ‘Journey with no map’ and ‘autonomy’ are ostensibly quite different ideas. However, the notion of embarking on a journey without a map invokes a sense of self-sufficiency and freedom that are features of autonomy.

##### (Un) structure

In interviews, people expressed that they had been part of a project that had no distinct boundaries and that even lacked a clear direction. This uncertainty necessarily influenced how the sessions were experienced:

*“There was always this part where we just didn't quite know, which made us a bit uncomfortable. At the same time, Daniel said, after the last time especially, “I like going because, when I'm there, my head doesn't hurt.”*

*(Rebecca)*

This observation from a partner captures the sense of not knowing, which was connected with a lack of structure. However, Rebecca similarly notes that whilst it made she and her husband ‘uncomfortable’ it may have been linked to a positive physical effect (his head didn't hurt) and so an increased sense of well-being. One participant with dementia also commented on the unstructured nature of the project:

*What was different was that it wasn't structured and that's what made the difference. It was more like going to a friend's house or spending some time with [a] friend*

*(Margaret)*

Margaret identifies that the distinctive quality of the sessions was a lack of structure but links this with the relaxed feeling of being with friends. She went on to say that this was quite different from art classes that she had previously attended. As Neil (a partner) noted: ‘*there is no format, there is no regime, it's free thinking and it flows very well’*. The interviewees' emphasis on the novelty of an unstructured, freer arts project, contrasts with the widely-held practice of arts groups with clearly delineated focus and an outcome oriented emphasis (discussed above).

##### Leadership

There were unexpected moments of leadership and creativity from people with dementia. As noted by Neil who attended every session with his wife Ruth who lives with dementia:

*I think that's the creative aspect of the whole thing… It's led by the people who you're trying to help, and if they were just sitting and being rather resistant to it all, that would show. But it's the opposite and everyone seems to benefit greatly*.

Often it was as a direct result of a ‘lull’ in the session, a feature often avoided in traditional arts projects, that a participant with dementia or partner began to dance or sing. It was noted by Margaret, who has dementia, that the group lacked a single leader ‘not one person in particular’. The ‘gap’ in leadership created an important space for those with dementia to exert agency. This was reflected on by Rebecca (a partner) who noted:

*I don't think there was much leadership, but I don't think that was the point…. the people who have a form of dementia have a space to be and have time for the others to make a space for their creation, …. In that sense, there was the leadership of letting it happen for each person, not letting one person take the whole space and time*

(Rebecca)

Further, video data revealed people with dementia leading activities, for example: by selecting a musical instrument and beginning to play it or using it as a prop, thus prompting others to engage with them and so re-directing the flow of the session. Leadership at these moments transferred between participants and was both a shared and negotiated phenomenon.

##### Unease

There were also moments of uncertainty, anxiety and even burden during sessions. After all, if there are few boundaries and individuals are invited to be creative agents this can also cause some discomfort. As Rebecca (partner) commented:

*I think there is this kind of awkwardness too of a situation like this.*

The video data depicts moments of uncomfortable silence in which the group sat waiting for ‘something’ to happen. Daniel who lives with dementia, said that to begin with he thought the sessions lacked a ‘rudder’ and his wife also noted that she thought he was sometimes ‘lost’ in terms of what role he should play. A lack of structure could therefore lead to an uneasy sense of disorganization. It is also recognised that within creative practice this type of ‘chaos’ can lead to important insights and developments, as evidenced, for example in the practice of devised theatre groups (Cavendish, 2015).

The co-creative process was by its nature risky, partly due to lack of expectations, but also because the testing of boundaries entailed the possibility that difficult emotions would be triggered. This is lucidly noted by one partner:

*‘…but people who do those things need to be aware that it could open up sore spots and things.’*

*(Rebecca)*

A sense of vulnerability was also noted by Rebecca. This was a complex state involving difficulties and also benefits. On the one hand, through sharing vulnerability the group became freer to co-create. However, sometimes people felt uncomfortably exposed:

*I can see that everybody has a tough time with lots of things. There is a vulnerability that is there too on top of everything else.*

*(Rebecca)*

Yet, Rebecca similarly recognised that being able to ‘peel’ off their outer layers enabled she and her husband Daniel (who has dementia) to be more involved in the co-creative process. The confrontation of vulnerability facilitated an exploration of challenging emotions and issues, such as anger and death e.g. as illustrated in the video data. These topics are often consciously avoided in arts projects for people with dementia due to concerns about arousing distress.

#### People together/connections

The central role of the group as part of the co-creative process was frequently noted in both the interview data as expressed in the theme ‘people together’ and in the theme from video data ‘connections’. These themes coincide and describe the sense of community that the co-creative sessions facilitated. The video and interview data reveal the group nurturing, caring for and attending to one another, for instance through gestures echoing one another's movements or stroking each other's backs, by inviting others to join a dance, or offering encouragement when improvising group songs.

##### Equality

The sense that everyone was ‘in it together’ was expressed in interviews and frequently observed in the video data. Thus Daniel (who lives with dementia) observed that his interest in the sessions was connected with their cooperative nature: ‘*It is interesting because people are cooperative*.’ This group cohesion also promoted a sense of equality. As Christopher (a partner) affirmed:

‘*everybody's equal, everybody plays their part*.’ Similarly, Eva who lives with dementia was prompted to reflect that the sessions had demonstrated fundamental shared humanity:

*No matter what, God created us all the same, we eat the same food, we drink water the same, so there's nothing different at all.*

*(Eva)*

The innately relational nature of the sessions was lucidly captured by people with dementia. Margaret, for instance, does not separate her contribution, from that of others. In response to a question about her specific involvement she answered:

*Well, it's just because it's all together. Everybody's important.*

She later stated:

*Well, yes because it's the whole group that makes a whole thing. That's why it's called ‘With All’, isn't it?*

The concept of shared ownership that exists within co-creativity (as outlined above) is evident in Margaret's response.

##### Shared experience & Friendship

The relevance of being part of a group with people who were having similar experiences was expressed by one partner (Neil):

*Because Ruth and I can empathise with other people in the same position as ourselves who might be at more advanced or less advanced stages of disability in terms of PCA (*Posterior cortical atrophy)*, Alzheimer's, and therefore one has less inhibitions and I think when Ruth is with other people in the group she has less inhibitions*.

Here Neil reflects on the shared experiences within the group and how for his wife, this liberates her. This was confirmed by Ruth, who noted:

*Yes. I felt very good. I felt very, what's the word, with it, with you and with everybody. I felt at ease. I felt happy*.

Another partner (Christopher) commented on how knowing that others in With All had partners with dementia, meant that friendships could be more easily formed. In turn, this created a particular type of community – one based on mutual empathy and an understanding of vulnerability that was easeful. As Eva (living with dementia) commented there was a sense of familiarity: ‘*as if you know them next door of your house’*.

##### Separate but together

However, the group was not always a contained or functioning whole. There were times when people were engaged in smaller, separate improvisations and yet there was nonetheless an impression of mutual involvement (as captured in video data).

There were several occasions when people chose to abruptly leave the session and were therefore perhaps asserting their separateness. Although, it is also noteworthy that when individuals re-entered the session they were seamlessly welcomed back into the group. The overall dynamics of the group were unaffected and it continued. There was a permissiveness that characterised the sessions that facilitated tolerance to dissenting behaviours as well as creative expression.

#### Engagement with art form/art as enabler

Engagement with art forms, in this case, music and dance was central to how co-creativity was enacted and experienced by participants. Instruments and movement were used by group members as largely non-verbal means of creating, connecting and performing. This emphasised the social and facilitative ethos of With All. As one participant with dementia noted:

*I liked the music …, and just listening to music and dancing, which I never believed I would get up and dance. I'm not very good at that sort of thing.*

(Alice)

The art forms were accessible, rejuvenating and nourishing, as one participant with dementia stated:

*Those people who have got bad experiences, whatever they are, when they hear music that they like they feel like water that has been dropped on them like a plant. When you let them drink, you feed them, they start to bloom, that's how I describe it*.

(Eva)

#### Play

The improvisational character of the sessions, coupled with a variety of unusual instruments and free style of dance ensured that the sessions were playful, for instance chime balls were used for bowling. Humour was also observed in video data, during shared moments of pleasure e.g. during a group dance or when Daniel (a participant with dementia) turned an instrument into a crown. In interview, Daniel stated:

*I like to make a fool of myself … Mainly because, I think, as I get older, people take it for granted, I won't.*

Here, he indicates that the co-creative sessions, in which he was able to play freely, enabled him to subvert expectations of himself as an older man.

The playfulness of the sessions and banter between participants has parallels with the practice of free improvisation, where musicians play in ways that seek to question the rules governing musical language (Bailey, 1992).

#### Confidence

In several cases, the With All sessions were linked with a discernable change in people's confidence. When considering how the sessions had affected Ruth, her husband observed:

*She's more outgoing, she's dancing and participating much more than otherwise, yes*

(Neil)

Equally, Alice who lives alone and has dementia, reflected that attending the groups had helped her become more organised and to make decisions in the rest of her life:

*I made the decision I have to have this eye done…*

(Alice)

As she later reflected:

*I think everything- you pick up more each week, and you realise, I mean I got up and did dancing today, which I've never done, and talking to people, and I liked the music. I think it's something that's increased as the weeks have gone on*

(Alice)

This indicates that the music and dance sessions, despite being characterised by an absence of ‘set’ activities, helped some people with dementia feel more in control and more ‘enabled’ in their daily lives. This supports the findings from the CWS concerning overall improvements in confidence.

#### Embodiment

There were many examples of participants using their bodies percussively within the space of the session. An example of embodiment included the use of musical instruments in non-traditional ways, such as a participant gently striking a tambourine against their head to create sound. This action was both a gestural and performative display, leading to a change in creative activity within the group, underlining the agency of participants with dementia. One participant with dementia noted the impact of improvised group dancing:

*You see they are too shy, but the music makes them move, they start to rock themselves and dancing and smiling…*

(Eva)

Here Eva comments on the connection between music and dance in the sessions and how this enabled participants to engage fully with the group through shared physical interactions.

### Canterbury well-being scale (CWS)

To measure wellbeing, the Canterbury Wellbeing Scale (CWS) was employed which is a subjective measure of wellbeing in individuals with early to middle stage dementia using visual analogue scales with five subscales (interested/bored, confident/not confident, happy/sad, well/unwell and optimistic/not optimistic). Before and after each session, individuals living with a dementia and their partners were asked to indicate how they felt at this very moment on each visual analogue subscale on a score from 0 to 100 with higher scores indicating a higher level of wellbeing (Johnson et al., 2017). A Visual Analogue Scale in the form of the CWS was used here since it has been found to be straightforward to use for individuals with cognitive decline and participants are able to complete the scale score themselves, thus showing high ecological validity and reliability (Johnson et al., 2017).

Quantitative data obtained from the CWS were analysed using SPSS version 24. Data was tested for normality in order to conduct parametric analyses. Paired samples *t*-tests were conducted to test for differences in scores obtained from persons living with a dementia and carers before and after each session. Data was analysed for the summed composite of the subscales for a composite wellbeing score. Although the research team were aware of the potential limitations of a scale, especially when applied to so few participants – it elicited some valuable results which together with the thematic analysis discussed above have contributed to an extended understanding of well-being. In addition, the scale had the unexpected benefit of provoking conversations and providing a segueway into each arts session.

Statistical analysis, for both composite and subscales of the CWS, showed an increase in wellbeing scores, denoting enhanced wellbeing, for people with dementia after the With All sessions compared to before the sessions. This difference is statistically significant after sessions one and three and a trend towards statistical significance can also be observed after session two after which scores also increase (see Table 2 below). Only findings related to the study aims are reported here due to space restrictions *(see appendix for further details)*.

**Table 2:** Mean increase in composite CWS scores before and after sessions for participants living with a dementia.

Session	Average increase	Significance (*p-*value)	Number of participants
Before and after session 1	61.75	0.026	4
Before and after session 2	32.25	0.066	4
Before and after session 3	20.8	0.022	5
Before and after session 4	−3.25	0.859	4

Note: significant differences are in bold (*p* < 0.05).

The confidence subscale for people with dementia, showed a significant increase in scores after session one. As with confidence, so a large significant increase in the well subscale score after the first session could be observed compared to before the session. Again, although not significantly, scores on the well subscale increased after sessions two and three and remained the same after session four. This finding corresponds with qualitative interviews, particularly participants stating how much they enjoyed the sessions but that they were sad they were finishing.

## Discussion

The findings support the role of co-creativity as an inclusive and equalizing approach. Co-creativity for this group was distinguished by a number of characteristic features, including empathic connections, a sense of equality and the generation of a safe space that enabled creative involvement and sharing.

The importance of working collaboratively with groups of people that are otherwise often excluded is being increasingly recognised (Humphries, 2017). There has been a growing awareness that people with dementia can play an active role in shaping their own services and can contribute in ways that are valid and enriching i.e. exerting agency (Bartlett & O'Connor, 2010; Ludwin & Capstick, 2017). However, there are a scarcity of ways of successfully including and working with people with dementia (Beard, 2004, Dowlen et al., 2017; Murphy, Jordan, Hunter, Cooney, & Casey, 2014). As noted by others this may in part be due to a lack of methodological ingenuity (Litherland & Capstick, 2014, p.410) and an absence of creatively inclusive responses (Shakespeare et al., 2017). Co-creativity, which emphasises non-hierarchical participation, represents a novel method for involving people with dementia in ways that are flexible and responsive.

In addition, our findings demonstrate that co-creativity affected experiences of well-being and agency of people with dementia and partners. The qualitative data together with the CWS data show that the With All sessions benefited the well-being of people with dementia, particularly increasing confidence and wellness. However, the CWS also demonstrated the difficulties of satisfactorily defining well-being with people with dementia. The quantitative nature of the CWS necessitates attributing a numerical value to a complex concept which may be regarded as reductive. Daniel (who lives with dementia) queried the CWS each week and in response to the question of ‘how well do you feel?’ insisted:

*Well, I mean I'm not well. I have to come to grips with that every day. But, I'm not feeling bad.*

However, the CWS encompasses multiple dimensions and in relation to With All demonstrated an increase in confidence amongst participants. This increase in confidence echoes the qualitative analysis of interview data. Using the CWS has resulted in a more robust understanding of the effect of the co-creative arts group on the multiple dimensions of well-being.

The findings indicate that well-being is not simply about feelings of increased happiness, interest or confidence. Neither is well-being something that necessarily equates with being in good physical or mental health. On the contrary, it is plausible that someone might be living with a chronic, degenerative condition (like Daniel) and yet consider themselves in a profound sense quite well. This is a complexity that has recently been acknowledged by the Lancet commission on global mental health (Patel et al., 2018:1562). To some extent, for people with dementia, well-being includes ill-being. Whilst the CWS plays a useful role in providing a snapshot of in the moment well-being, there is no measure of well-being that captures the ‘ill-being’ that is a necessary part of life with dementia.

The co-creative sessions helped enhance well-being by allowing participants to be vulnerable and share this with others. This is not necessarily either comfortable or easy and was expressed as unease. In order to express difficult, confronting emotions, we need to have agency, to be enabled to find our voice in our own idiosyncratic language to communicate how our experience feels. The co-creative process can facilitate this through play (Brown, 2010) and by providing moments of catharsis and release. The co-creative sessions involved periods of silence or pause. These may involve discomfort but also opened the opportunity and space for agency and creative expression. Well-being then might be most fully understood as dependent on feeling agential.

Agency for people with dementia, in the context of co-creative sessions was partly demonstrated in the moments when people with dementia assumed leadership of the session. This provides an interesting counterpoint to prevailing views that people with dementia are dependent and require guidance. At times, agency might also involve apparent passivity, not actively leading a dance or tune but nonetheless remaining part of the group and maintaining its rhythm. In line with Kontos et al. (2017) agency was embodied i.e.: physically enacted through gesture, movement (leaving a room), music and dance but it is also cognitive – participants considered and were able to reflect on their varying levels of engagement. Agency and well-being according to our findings are perhaps best comprehended as relational, and constitutive of an ongoing practice, rather than completed states, and which contain their opposites (ill-being and passivity).

Thus, neither well-being or agency can be understood as having a directional/linear progression for people with dementia, they are therefore hard to measure in terms of before and after. Above all, well-being and agency are refracted by our wider context and by our awareness of our own finitude. The unique role of co-creativity for expanding our understanding of both well-being and agency is as a process that does not rely on pre-determined co-ordinates. Rather people are involved as interdependent, creative agents with equal potential to explore (and get lost) together.

## Strengths and limitations

This study is the first to consider the notions of co-creativity, well-being and agency from the perspectives of those living with dementia. The use of combined video, interview and CWS data ensured that rich and nuanced accounts of participant experiences were captured. The study had a number of limitations however. The sessions were attended by a small group who were demographically similar: five people with dementia and three partners who all lived in London and were culturally educated. The study is therefore prone to bias of chance and social desirability. In addition, the specific context of the study (the Wellcome Collection) is not readily known or accessible to most people with dementia. There may be difficulties of replication due to complications with sharing the methodology with others and its possible dependency on particular artists experienced in working in a co-creative way. The use of mixed research methods as described above, were crucial for capturing the experiences of people with dementia and their partners. However, paradoxically these necessitated an uneven relationship between researchers and ‘participants’ which undermined the co-creative process and involved further complexity. With All, like most arts projects, was tightly time-limited due to funding constraints and may therefore create dependency and then be withdrawn.

Above all, this paper is not co-written by someone living with dementia, although there were several meetings after the project ended, where all involved were invited to contribute to the research process and findings. Future studies investigating and deploying co-creative methods with people with dementia should be larger, more diverse and more courageous in their use of innovative research methods.

## Conclusion

This study demonstrated the potential for inclusive and supportive methods of deploying the arts co-creatively with people with dementia. It represents an innovative approach for exploring the arts alongside people with dementia and their partners, rather than ‘giving’ the arts directively. People with dementia and their partners valued and benefitted from this approach, although uncomfortable elements of co-creativity were also identified.

Co-creativity has helped to extend our understanding of the complex concepts of well-being and agency in relation to dementia.

Future research should replicate the co-creative approach in different contexts and with diverse demographics to investigate wider benefits of co-creativity in dementia care. The findings suggest that well-being and agency for people with dementia should be reconsidered. The co-creative arts offer possibilities for agential engagement that may advance equalities based approaches that recognise and develop the citizenship and human rights of people with dementia.

## Appendix A Supplementary data

Supplementary material Image 1

## References

[bb0005] Aesop & BOP (2018). Active ingredients: The Aesop planning and evaluation model for arts with a social purpose. http://www.ae-sop.org/2018/09/14/aesop-launches-active-ingredients/.

[bb0010] All-Party Parliamentary Group on Arts, Health and Wellbeing (APPGAHW) (2017). Creative health: The arts for health and wellbeing. https://www.artshealthandwellbeing.org.uk/appg-inquiry/.

[bb0015] Aquilina C., Hughes J.C., Hughes J., Louw S.J., Sabat S.R. (2005). The return of the living dead: agency lost and found?. Dementia, mind, meaning and the person.

[bb9005] Bailey D. (1992). Improvisation: Its nature and practice.

[bb0025] Bartlett R., O'Connor D. (2010). Broadening the dementia debate, towards social citizenship.

[bb0030] Beard R. (2004). Advocating voice: Organisational, historical and social milieu of the Alzheimer's disease movement. Sociology of Health & Illness.

[bb0040] Bellass S., Balmer A., May V., Keady J., Buse C., Capstick A., …Hodgson, J. (2018). Broadening the debate on creativity and dementia: A critical approach. Dementia.

[bb0045] Boyle G. (2014). Recognising the agency of people with dementia. Disability & Society.

[bb0050] Braun V., Clarke V. (2006). Using thematic analysis in psychology. Qualitative Research in Psychology.

[bb0055] Brown S. (2010). Play: How it shapes the brain, opens the imagination, and invigorates the soul.

[bb0060] Burke L. (2017). Imagining a future without dementia: Fictions of regeneration and the crises of work and sustainability. Palgrave Communications.

[bb0065] Camic P.M., Crutch S., Murphy C., Firth N., Harding E., Harrison C., Zeilig H. (2018). Conceptualising and understanding artistic creativity in the dementias: Interdisciplinary approaches to research and practice. Frontiers in Psychology.

[bb0070] Camic P.M., Crutch S., Zeilig H. (2018). The arts and dementia: Emerging directions for theory, research and practice. Dementia.

[bb0075] Cavendish D. (2015, March 8). Simon McBurney on devised theatre: ‘It's absolutely petrifying!’. The Telegraph.

[bb9000] Chung P.Y.F., Ellis-Hill C., Coleman P. (2017). Supporting activity engagement by family carers at home: maintenance of agency and personhood in dementia. International Journal of Qualitative Studies on Health and Well-being.

[bb0090] Crowe S., Cresswell K., Robertson A., Huby G., Avery A., Sheikh A. (2011). The case study approach. BMC Medical Research Methodology.

[bb0095] Crutch S.J., Isaacs R., Rossor M.N. (2001). Some workmen can blame their tools: Artistic change in an individual with Alzheimer's disease. The Lancet.

[bb0100] Derry S.J. (2007). Guidelines for video research in education. https://drdc.uchicago.edu/what/video-research-guidelines.pdf.

[bb0105] Dowlen R., Keady J., Milligan C., Swarbrick C., Ponsillo N., Geddes L., Riley B. (2017). The personal benefits of musicking for people living with dementia: A thematic synthesis of the qualitative literature. Arts & Health.

[bb0125] Gross S.M., Danilova D., Vandehey M.A. (2015). Creativity and dementia: Does artistic activity affect well-being beyond the art class?. Dementia.

[bb0130] Hellström I., Nolan M., Lundh U. (2005). ‘We do things together’: A case study of ‘couplehood’ in dementia. Dementia.

[bb0140] Hughes J.C., Louw S.J., Sabat S.R., Hughes J., Louw S., Sabat S.R. (2005). Seeing whole. Dementia: Mind, meaning and the person.

[bb0145] Hughes J.C., Williamson T. (2019). The dementia manifesto: Putting values-based practice to work.

[bb0150] Humphries B. (2017). Re-thinking social research anti-discriminatory approaches in research methodology.

[bb0155] Jaworska A. (1999). Respecting the margins of agency: Alzheimer's patients and the capacity to value. Philosophy & Public Affairs.

[bb0160] Jennings B., Kittay E.F., Carlson L. (2010). Agency and moral relationship in dementia. Cognitive disability and its challenge to moral philosophy.

[bb0165] Johnson J., Culverwell A., Hulbert S., Robertson M., Camic P.M. (2017). Museum activities in dementia care: Using visual analog scales to measure subjective wellbeing. Dementia.

[bb0175] Kaufmann E.G., Engel S.A. (2016). Dementia and well-being: A conceptual framework based on Tom Kitwood's model of needs. Dementia.

[bb0185] Kitwood T., Bredin K. (1992). Towards a theory of dementia care: Personhood and well-being. Ageing and Society.

[bb0195] Kolanowski A., Buettner L. (2008). Prescribing activities that engage passive residents. An innovative method. Journal of Gerontological Nursing.

[bb0200] Kontos P., Miller K.-L., Kontos A.P. (2017). Relational citizenship: Supporting embodied selfhood and relationality in dementia care. Sociology of Health & Illness.

[bb0205] Krishna V.V. (2014). Changing social relations between science and society: Contemporary challenges. Science, Technology and Society.

[bb0215] Levinson J. (2015). Musical concerns: Essays in philosophy of music.

[bb0220] Litherland R., Capstick A., Downs M., Bowers B. (2014). Involving people with dementia in service evaluation. Excellence in Dementia Care.

[bb0225] Ludwin K., Capstick A. (2017). Ethnography in dementia care research: Observations on ability and capacity. SAGE research methods cases - ethnography.

[bb0235] Mobjörk M. (2010). Consulting versus participatory transdisciplinarity: A refined classification of transdisciplinary research. Futures.

[bb0240] Murphy K., Jordan F., Hunter A., Cooney A., Casey D. (2014). Articulating the strategies for maximising the inclusion of people with dementia in qualitative research studies. Dementia.

[bb0245] National Institute for Health and Care Excellence (NICE) (2015). Older people: independence and mental wellbeing, (NICE Guideline 32). https://www.nice.org.uk/guidance/ng32.

[bb0250] O'Connor D., Mann J., Wiersma E. (2018). Stigma, Discrimination and agency: diagnostic disclosure as an everyday practice shaping social citizenship. Journal of Aging Studies.

[bb0255] Office for National Statistics (2018). Personal well-being in the UK: July 2017 to June 2018. https://www.ons.gov.uk/peoplepopulationandcommunity/wellbeing/bulletins/measuringnationalwellbeing/july2017tojune2018.

[bb0260] Osman S.E., Tischler V., Schneider J. (2016). ‘Singing for the Brain’: A qualitative study exploring the health and well-being benefits of singing for people with dementia and their carers. Dementia.

[bb0265] Palmiero M., Di Giacomo D., Passafiume D. (2012). Creativity and dementia: A review. Cognitive Processing.

[bb0270] Patel V., Saxena S., Lund C., Thornicroft G., Baingana F., Bolton P., UnÜtzer J. (2018). The lancet commission on global mental health and sustainable development. The Lancet.

[bb0285] Russel, G. M., Kelly, N. H. (2002) Research as interacting dialogic processes: Implications for reflexivity. Forum: Qualitative social research, [S.l.], v. 3, n. 3, sep. 2002. ISSN 1438–5627. Available at: <http://www.qualitative-research.net/index.php/fqs/article/view/831>. Date accessed: 27 oct. 2017. doi:10.17169/fqs-3.3.831.

[bb0295] Saldaña J. (2016). The coding manual for qualitative researchers.

[bb0300] Sauer P.E., Fopma-Loy J., Kinney J.M., Lokon E. (2014). “It makes me feel like myself”: Person-centred versus traditional arts activities for people with dementia. Dementia.

[bb0305] Schlosser M., Zalta E.N. (2015). Agency. The stanford encyclopedia of philosophy (fall 2015).

[bb0310] Schmöelz A. (2017). On co-creativity in playful classroom activities. In De Gruyter Open. https://www.researchgate.net/publication/318655679_On_Co-Creativity_in_Playful_Classroom_Activities.

[bb0315] Seligman M. (2012). Flourish: A visionary new understanding of happiness and well-being.

[bb0320] Shakespeare T., Zeilig H., Mittler P. (2017). Rights in mind: Thinking differently about dementia and disability. Dementia.

[bb0325] Shin D.C., Johnson D.M. (1978). Avowed happiness as an overall assessment of the quality of life. Social Indicators Research.

[bb0330] Small C. (1998). Musicking: The meanings of performing and listening.

[bb0335] Strohmaier S., Camic C. (2017, November 24). Conceptualising what we mean by ‘wellbeing in the dementias. Paper presented at the Royal Society for public health (RSPH) conference titled Powerful Partners: Advancing dementia care through the arts and sciences.

[bb0345] Taylor C. (1992). Sources of the self: The making of the modern identity.

[bb0355] Toomey A. (2015). ‘Inter-and transdisciplinary research : A critical perspective’, in.

[bb0370] Windle G., Joling K., Howson-Griffiths T., Woods B., Jones C.H., van de Ven P., Parkinson C. (2017). The impact of a visual arts programme on quality of life, communication and well-being of people living with dementia: A mixed-methods longitudinal investigation. International Psychogeriatrics.

[bb0375] Yin R.K. (1994). Case study research: Design and methods.

[bb0380] Young R., Camic P.M., Tischler V. (2016). The impact of community-based arts and health interventions on cognition in people with dementia: A systematic literature review. Aging & Mental Health.

[bb0385] Zeilig H. (2014). Dementia as a cultural metaphor. Gerontologist.

[bb0390] Zeilig H., West J., van der Byl Williams M. (2018). Co-creativity: Possibilities for using the arts with people with a dementia. Quality in Ageing and Older Adults.

