# Supplementary material for: Co-creativity, well-being and agency: A case study analysis of a co-creative arts group for people with dementia
Source: J Aging Stud. 2019 Jun;49:16–24. doi: 10.1016/j.jaging.2019.03.002 (PMC6597952; doi:10.1016/j.jaging.2019.03.002)
Supplement: Supplementary file 1 — Supplementary material [file mmc1.docx]

Results on the CWS scale for the With All sessions data

For each of the different data collection time points, the average scores on the composite CWS scale as well as each of the subscales of the CWS were compared before the programme and after the programme using a t-test.

The data was analysed when including all participants as well as with three separate groups, namely participants who were artists, participants who were living with a dementia and participants who were carers.

In this report, the results when all participants were included in the analysis are listed first, followed by the results separately for each participant group.

Results when all participants were included

**Composite CWS scale**

For the composite scale, there was a statistically significant increase in average scores after the sessions compared to before the sessions at data collection points two and three. Although there was an increase in composite CWS scores at times one and four, this was not statistically significant. When combining all pre-session and all post-session scores, there was a statistically significant increase in CWS scores.

See Table 1 for average increase in CWS scores for the four time points data was collected as well as for overall increase from before to after the sessions.

Table 1:

*Mean increase in composite CWS scores before and after sessions*

| **Session** | **Average increase** | **Significance**  **(*p-*value)** | **Number of participants** |
| --- | --- | --- | --- |
| Before and after session 1 | 38.43 | 0.062 | 7 |
| Before and after session 2 | **35** | **0.003** | 8 |
| Before and after session 3 | **28.33** | **<0.001** | 9 |
| Before and after session 4 | 24.11 | 0.124 | 9 |
| Before and after all sessions | **102.8** | **0.003** | 33 |

*Note: significant differences are in* ***bold (p<0.05)***

Figure 1 displays the average composite scale scores across the four data collection points.

Figure 1:

*Average total scale scores across the four data collection points*

**
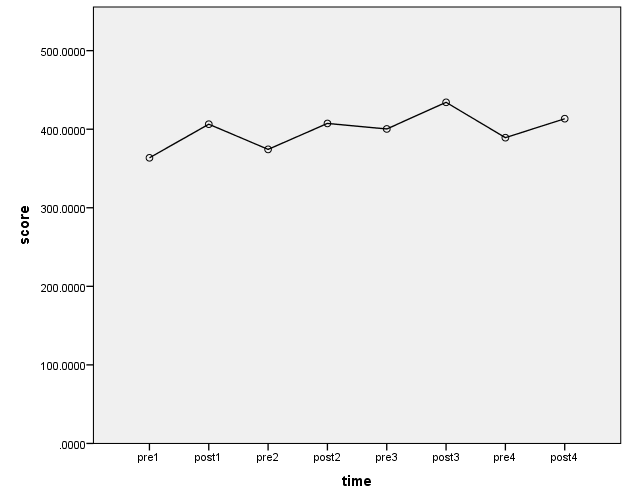
**

**CWS by subscales**

**Interest**

For the interested-bored subscale, there was not a statistically significant increase in average scores after the sessions compared to before the sessions on any time points of data collection. Although this was not significant, there was an increase in average interest scores at times one to three, but not at time four.

Table 2*:*

*Mean increase in interest scores before and after sessions*

| **Session** | **Average increase** | **Significance (*p*-value)** |
| --- | --- | --- |
| Before and after session 1 | 4.71 | 0.469 |
| Before and after session 2 | 1.63 | 0.637 |
| Before and after session 3 | 6.11 | 0.102 |
| Before and after session 4 | 0.89 | 0.819 |

*Note: significant differences are in* ***bold (p<0.05)***

**Confidence**

For the confident-not confident subscale, there was a statistically significant average increase of 6.33 in scores after the sessions compared to before the sessions for the third time point of data collection, but not for time points one, two and four. Although not significant, there was an increase in average confidence scores after the intervention compared to before the intervention at those three time points as well.

Table 3*:*

*Mean increase in confidence scores before and after sessions*

| **Session** | **Average increase** | **Significance (*p*-value)** |
| --- | --- | --- |
| Before and after session 1 | 8.14 | 0.106 |
| Before and after session 2 | 3.63 | 0.283 |
| Before and after session 3 | **6.33** | **0.005** |
| Before and after session 4 | 6.33 | 0.114 |

*Note: significant differences are in* ***bold (p<0.05)***

**Optimism**

For the optimistic-not optimistic subscale, there was a statistically significant average increase of 10.13 in scores after the sessions compared to before the sessions for the second time point of data collection, but not for the time points one, three and four. Although not significant, there was an increase in average optimism scores after the intervention compared to before the intervention at those three time points as well.

Table 4*:*

*Mean increase in optimism scores before and after sessions*

| **Session** | **Average increase** | **Significance (*p*-value)** |
| --- | --- | --- |
| Before and after session 1 | 5.71 | 0.434 |
| Before and after session 2 | **10.13** | **0.017** |
| Before and after session 3 | 4.11 | 0.11 |
| Before and after session 4 | 4.44 | 0.154 |

*Note: significant differences are in* ***bold (p<0.05)***

**Happiness**

For the happy-sad subscale, there was a statistically significant increase in average scores after the sessions compared to before the sessions for time points one and two, but not for three and four. See Table 5 for average increase in happiness scores at time points one and two. However, although the difference in average happiness scores was not statistically significant after sessions three and four, there was an increase in happiness scores at these time points as well.

Table 5*:*

*Mean increase in happiness scores before and after sessions*

| **Session** | **Average increase** | **Significance (*p*-value)** |
| --- | --- | --- |
| Before and after session 1 | **18.22** | **0.05** |
| Before and after session 2 | **12** | **0.029** |
| Before and after session 3 | 6.33 | 0.126 |
| Before and after session 4 | 9.22 | 0.123 |

*Note: significant differences are in* ***bold (p<0.05)***

**Well**

For the well-unwell subscale, there was a statistically significant increase in average scores after the sessions compared to before the sessions for time points one, two and three but not for time point four. Although this was not significant, there was an increase in average well scores after session four as well.

See Table 6 for average increase in well scores.

Table 6:

*Mean increase in well scores before and after sessions*

| **Session** | **Average increase** | **Significance (*p*-value)** |
| --- | --- | --- |
| Before and after session 1 | **10.56** | **0.033** |
| Before and after session 2 | **12.11** | **0.018** |
| Before and after session 3 | **5.44** | **0.045** |
| Before and after session 4 | 5 | 0.371 |

*Note: significant differences are in* ***bold (p<0.05)***

Results divided by participant groups

Artists

**Composite CWS scale**

Overall, there was an average increase of 112 (*p*=0.129) on the composite CWS scores for the artist population, though this was not statistically significant (*p*>0.05).

When looking at the four time points separately, although there was an increase in CWS scores at all time points, this was not a statistically significant difference for the artist participants.

Table 7:

*Mean increase in composite CWS scores before and after sessions for artist population group*

| **Session** | **Average increase** | **Significance**  **(*p-*value)** | **Number of participants** |
| --- | --- | --- | --- |
| Before and after session 1 | 7.33 | 0.802 | 3 |
| Before and after session 2 | 20 | 0.126 | 2 |
| Before and after session 3 | 45.5 | 0.077 | 2 |
| Before and after session 4 | 64 | 0.383 | 2 |

*Note: significant differences are in* ***bold (p<0.05)***

**Interest**

For the interest subscale, there was no statistically significant difference before and after the sessions for the artist participants.

Table 8*:*

*Mean increase in interest scores before and after sessions for artist participant group*

| **Session** | **Average increase** | **Significance (*p*-value)** |
| --- | --- | --- |
| Before and after session 1 | 4.33 | 0.669 |
| Before and after session 2 | -3.5 | 0.258 |
| Before and after session 3 | 10 | 0.5 |
| Before and after session 4 | 4 | 0.823 |

*Note: significant differences are in* ***bold (p<0.05)***

**Confidence**

For the confidence subscale, there was a statistically significant average increase of 10.5 in scores after the sessions compared to before the sessions for the third time point of data collection, but not for time points one, two and four for the artist participants.

Table 9*:*

*Mean increase in confidence scores before and after sessions for artist participant group*

| **Session** | **Average increase** | **Significance (*p*-value)** |
| --- | --- | --- |
| Before and after session 1 | -2.67 | 0.56 |
| Before and after session 2 | -4 | 0.295 |
| Before and after session 3 | **10.5** | **0.03** |
| Before and after session 4 | 7.5 | 0.656 |

*Note: significant differences are in* ***bold (p<0.05)***

**Optimism**

For the optimism subscale, there was no statistically significant difference before and after the sessions for the artist participants.

Table 10*: Mean increase in optimism scores before and after sessions for artist participant group*

| **Session** | **Average increase** | **Significance (*p*-value)** |
| --- | --- | --- |
| Before and after session 1 | -6.67 | 0.63 |
| Before and after session 2* | 0 | 1 |
| Before and after session 3 | 5 | 0.5 |
| Before and after session 4 | 12.5 | 0.126 |

*Note: significant differences are in* ***bold (p<0.05),*** **scores remained the same before and after this session.*

**Happiness**

For the happiness subscale, there was no statistically significant difference before and after the sessions for the artist participants.

Table 11*:*

*Mean increase in happiness scores before and after sessions for artist participant group*

| **Session** | **Average increase** | **Significance (*p*-value)** |
| --- | --- | --- |
| Before and after session 1 | 14 | 0.139 |
| Before and after session 2 | 16 | 0.118 |
| Before and after session 3 | 18 | 0.354 |
| Before and after session 4 | 30 | 0.374 |

*Note: significant differences are in* ***bold (p<0.05)***

**Well**

For the well subscale, there was no statistically significant difference before and after the sessions for the artist participants. There was no data available before and after session 4 for the artist population group.

Table 12*:*

*Mean increase in well scores before and after sessions for artist participant group*

| **Session** | **Average increase** | **Significance (*p*-value)** |
| --- | --- | --- |
| Before and after session 1 | -1.67 | 0.789 |
| Before and after session 2 | 11.5 | 0.527 |
| Before and after session 3 | 1.5 | 0.5 |
| Before and after session 4 | N/A | N/A |

*Note: significant differences are in* ***bold (p<0.05)***

Living with a dementia

**Composite CWS score**

There was no statistically significance in composite CWS scores for the living with dementia participants. However, although this was not significant, there was an increase in scores from before to after the sessions (mean increase= 96.67; *p*=0.065).

When looking at the time points separately, there was a statistically significant average increase in scores on the CWS at time points one and three. Although this was not statistically significant, there was an increase in average CWS scores at time point two and an overall decrease at time point four, although this was again, not statistically significant. (see Table 13 for the scores for each of the time points).

Table 13:

*Mean increase in composite CWS scores before and after sessions for participants living with a dementia*

| **Session** | **Average increase** | **Significance**  **(*p-*value)** | **Number of participants** |
| --- | --- | --- | --- |
| Before and after session 1 | **61.75** | **0.026** | 4 |
| Before and after session 2 | 32.25 | 0.066 | 4 |
| Before and after session 3 | **20.8** | **0.022** | 5 |
| Before and after session 4 | -3.25 | 0.859 | 4 |

*Note: significant differences are in* ***bold (p<0.05)***

**Interest**

For the interest subscale, there was no statistically significant difference before and after the sessions for the participants living with a dementia.

Table 14*:*

*Mean increase in interest scores before and after sessions for participants living with a dementia*

| **Session** | **Average increase** | **Significance (*p*-value)** |
| --- | --- | --- |
| Before and after session 1 | 5 | 0.638 |
| Before and after session 2 | 5 | 0.495 |
| Before and after session 3 | 5 | 0.374 |
| Before and after session 4 | 5.5 | 0.432 |

*Note: significant differences are in* ***bold (p<0.05)***

**Confidence**

For the confidence subscale, there was a statistically significant average increase of 16.25 in scores after the sessions compared to before the sessions for the first time point of data collection, but not for time points two, three and four for the participants living with a dementia. Although not significant, there was an increase in average interest scores at these time points as well for the participants living with a dementia.

Table 15*:*

*Mean increase in confidence scores before and after sessions for participants living with a dementia*

| **Session** | **Average increase** | **Significance (*p*-value)** |
| --- | --- | --- |
| Before and after session 1 | **16.25** | **0.007** |
| Before and after session 2 | 1.75 | 0.581 |
| Before and after session 3 | 5.2 | 0.081 |
| Before and after session 4 | 1.5 | 0.786 |

*Note: significant differences are in* ***bold (p<0.05)***

**Optimism**

For the optimism subscale, there was no statistically significant difference before and after the sessions for the participants living with a dementia.

Table 16*:*

*Mean increase in optimism scores before and after sessions for participants living with a dementia*

| **Session** | **Average increase** | **Significance (*p*-value)** |
| --- | --- | --- |
| Before and after session 1 | 15 | 0.058 |
| Before and after session 2 | 12.75 | 0.054 |
| Before and after session 3 | 3.4 | 0.408 |
| Before and after session 4 | -1.25 | 0.789 |

*Note: significant differences are in* ***bold (p<0.05)***

**Happiness**

For the happiness subscale, there was no statistically significant difference before and after the sessions for the participants living with a dementia.

Table 17*:*

*Mean increase in happiness scores before and after sessions for participants living with a dementia*

| **Session** | **Average increase** | **Significance (*p*-value)** |
| --- | --- | --- |
| Before and after session 1 | 10.5 | 0.074 |
| Before and after session 2 | 3.75 | 0.547 |
| Before and after session 3 | 2 | 0.621 |
| Before and after session 4 | 2 | 0.514 |

*Note: significant differences are in* ***bold (p<0.05)***

**Well**

For the well subscale, there was a statistically significant average increase of 15 in scores after the sessions compared to before the sessions for the first time point of data collection, but not for the remaining time points for the participants living with a dementia.

Table 18*:*

*Mean increase in well scores before and after sessions for participants living with a dementia*

| **Session** | **Average increase** | **Significance (*p*-value)** |
| --- | --- | --- |
| Before and after session 1 | **15** | **0.018** |
| Before and after session 2 | 9 | 0.186 |
| Before and after session 3 | 5.2 | 0.081 |
| Before and after session 4* | 0 | 1 |

*Note: significant differences are in* ***bold (p<0.05);*** **scores remained the same before and after this session.*

Carers

**Composite CWS score**

For carers, there was no data to calculate average difference before and after all sessions combined since there was no data available for the carer participants before and after session one.

When looking at the three time points separately, although there was an increase in CWS scores at the three time points where data was available, this was not a statistically significant difference for the carer participants.

Table 19*:*

*Mean increase in composite CWS scores before and after sessions for carer participants*

| **Session** | **Average increase** | **Significance**  **(*p-*value)** | **Number of participants** |
| --- | --- | --- | --- |
| Before and after session 1 | N/A | N/A | N/A |
| Before and after session 2 | 55.5 | 0.163 | 2 |
| Before and after session 3 | 30 | 0.205 | 2 |
| Before and after session 4 | 34 | 0.06 | 3 |

*Note: significant differences are in* ***bold (p<0.05)***

**Interest**

For the interest subscale, there was no statistically significant difference before and after the sessions for the carer participants. Carer data was only available before and after sessions three and four for the interest subscale.

Table 20*:*

*Mean increase in interest scores before and after sessions for carer participants*

| **Session** | **Average increase** | **Significance (*p*-value)** |
| --- | --- | --- |
| Before and after session 1 | N/A | N/A |
| Before and after session 2 | N/A | N/A |
| Before and after session 3 | 5 | 0.5 |
| Before and after session 4 | 2 | 0.423 |

*Note: significant differences are in* ***bold (p<0.05)***

**Confidence**

For the confidence subscale, there was no statistically significant difference before and after the sessions for the carer participants. Carer data was not available before and after session 1.

Table 21*:*

*Mean increase in confidence scores before and after sessions for carer participants*

| **Session** | **Average increase** | **Significance (*p*-value)** |
| --- | --- | --- |
| Before and after session 1 | N/A | N/A |
| Before and after session 2 | 15 | 0.205 |
| Before and after session 3 | 5 | 0.5 |
| Before and after session 4 | 12 | 0.102 |

*Note: significant differences are in* ***bold (p<0.05)***

**Optimism**

For the optimism subscale, there was no statistically significant difference before and after the sessions for the carer participants. Carer data was not available before and after session 1.

Table 22*:*

*Mean increase in optimism scores before and after sessions for carer participants*

| **Session** | **Average increase** | **Significance (*p*-value)** |
| --- | --- | --- |
| Before and after session 1 | N/A | N/A |
| Before and after session 2 | 15 | 0.205 |
| Before and after session 3 | 5 | 0.5 |
| Before and after session 4 | 6.67 | 0.184 |

*Note: significant differences are in* ***bold (p<0.05)***

**Happiness**

For the happiness subscale, there was no statistically significant difference before and after the sessions for the carer participants. Carer data was not available before and after session 1.

Table 23*:*

*Mean increase in happiness scores before and after sessions for carer participants*

| **Session** | **Average increase** | **Significance (*p*-value)** |
| --- | --- | --- |
| Before and after session 1 | N/A | N/A |
| Before and after session 2 | 20.33 | 0.17 |
| Before and after session 3 | 5 | 0.5 |
| Before and after session 4 | 5 | 0.225 |

*Note: significant differences are in* ***bold (p<0.05)***

**Well**

For the well subscale, there was a statistically significant average increase of 8.33 in scores after the sessions compared to before the sessions for the fourth time point of data collection, but not for the remaining time points for the carer participants.

Table 24*:*

*Mean increase in well scores before and after sessions for carer participants*

| **Session** | **Average increase** | **Significance (*p*-value)** |
| --- | --- | --- |
| Before and after session 1 | 20 | 0.295 |
| Before and after session 2 | 16.67 | 0.199 |
| Before and after session 3 | 10 | 0.5 |
| Before and after session 4 | **8.33** | **0.038** |

*Note: significant differences are in* ***bold (p<0.05)***
